# *Hugonella massiliensis* gen. nov., sp. nov., genome sequence, and description of a new strictly anaerobic bacterium isolated from the human gut

**DOI:** 10.1002/mbo3.458

**Published:** 2017-03-21

**Authors:** Ziena Elsawi, Amadou Hamidou Togo, Mamadou Beye, Grégory Dubourg, Claudia Andrieu, Nicholas Armsrtong, Magali Richez, Fabrizio di Pinto, Fadi Bittar, Noémie Labas, Pierre‐Edouard Fournier, Didier Raoult, Saber Khelaifia

**Affiliations:** ^1^ Unité de Recherche sur les Maladies Infectieuses et Tropicales Emergentes CNRS (UMR 7278) IRD (198) INSERM (U1095) AMU (UM63) Institut Hospitalo‐Universitaire Méditerranée‐Infection Faculté de médecine Aix‐Marseille Université Marseille France; ^2^ Special Infectious Agents Unit King Fahd Medical Research Center King Abdulaziz University Jeddah Saudi Arabia

**Keywords:** culturomics, *Hugonella massiliensis* sp. nov., obesity, taxonogenomic

## Abstract

The human gut is composed of a large diversity of microorganisms, which have been poorly described. Here, using culturomics, a new concept based on the variation in culture conditions and MALDI‐TOF MS identification, we proceed to explore the microbial diversity of the complex ecosystem of the human gut. Using this approach, we isolated strain AT8^T^ (=CSUR P2118 =  DSM 101782) from stool specimens collected from a 51‐year‐old obese French woman. Strain AT8^T^ is a strictly anaerobic, nonmotile, nonspore‐forming gram‐positive coccus that do not exhibit catalase and oxidase activities. 16S rDNA‐based identification of strain AT8^T^ demonstrated 92% gene sequence similarity with *Eggerthella lenta* DSM 2243, the phylogenetically closed validly named type species. Here, we present a set of features for the strain AT8^T^ and the description of its complete genome sequence and annotation. The 2,091,845 bp long genome has a G+C content of 63.46% and encodes1,849 predicted genes; 1,781 were protein‐coding genes, and 68 were RNAs. On the basis of the characteristics reported here, we propose the creation of a new bacterial genus *Hugonella* gen. nov.*,* belonging to the *Eggerthellaceae* family and including *Hugonella massiliensis* gen. nov., sp. nov.*,* strain AT8^T^ as the type strain.

## 1 Introduction

The human gut harbors a complex bacterial community known as microbiota. However, this ecosystem remains incompletely characterized and its diversity poorly described (Eckburg et al., 2005; Lozupone, Stombaugh, Gordon, Jansson, & Knight, 2012). Culturomics concepy was recently proposed as a new alternative to explore this ecosystem and enriches the human microbiota repertoire. This method is based on the large variation in culture conditions and the use of rapid bacterial identification methods such as matrix‐assisted laser desorption/ionization time‐of‐flight mass spectrometry (MALDI‐TOF) and 16S rRNA gene amplification and sequencing of the colonies (Lagier et al., 2015). Traditionally, several parameters were used to identify and define a new bacterial species including 16S rRNA gene sequencing and phylogeny, genomic diversity of the G+C content, DNA‐DNA hybridization (DDH) intensive phenotypic, and chemotaxonomic characterization (Ramasamy, Mishra, Lagier, Padhmanabhan, & Rossi, 2014; Welker & Moore, 2011). Nevertheless, some limits have been appeared notably because the cutoff values vary dramatically between species and genera (Rosselló‐Móra, 2006). So in order to describe new bacterial species, we recently proposed a new method named taxonogenomics, which includes both genomic analysis and proteomic information obtained by MALDI‐TOF analysis (Ramasamy et al., 2014). Using culturomics techniques (Lagier et al., 2015), we herein isolated strain AT8^T^ from a stool specimen of a 51‐year‐old obese French woman (BMI 44.38 kg/m^2^). Here, we present a classification and a set of characteristics of strain AT8^T^ together with the description of its complete genome sequencing and annotation that allowed us to describe them as the first representative of a new bacterial genus classified into *Eggerthellaceae* family within the phylum *Actinobacteria*. The *Eggerthellaceae* family contains nine different genera, *Adlercreutzia*,* Asaccharobacter*,* Cryptobacterium*,* Denitrobacterium*,* Eggerthella, Enterorhabdus*,* Gordonibacter*,* Paraeggerthella,* and *Slackia* (Gupta et al. 2006). The species of this family are strictly anaerobic cocci and they do not form spores (Gupta et al. 2006).

## 2 Methods and Materials

### 2.1 Ethics and sample collection

The stool sample was collected from a 51‐year‐old obese French woman (BMI 44.38 kg/m^2^; weight 108 kg, 1.56 meters in height) in January 2012. Written consent was obtained from the patient at the Nutrition, Metabolic Disease and Endocrinology service, at La Timone Hospital, (Marseille, France). The study and the consent procedures were approved by the local IFR 48 ethics committee, under consent number 09‐022, 2010. The stool sample was stored at −80°C after collection.

### 2.2 Isolation of the strain

Strain AT8^T^ was isolated in June 2015 by anaerobic culture. Approximately, 1 g of stool specimen was inoculated anaerobically in an anaerobic blood culture bottle supplemented with 5% (v/v) sheep blood and 5% (v/v) rumen fluid. pH was adjusted at 7.5 using KOH solution (10%) and the blood culture bottle was incubated at 37°C for 3 days. After 3 days incubation, subcultures were done on solid medium consisting of Columbia agar supplemented with 5% sheep blood and incubated anaerobically for 48 hr. All growing colonies were picked several times to obtain pure cultures.

### 2.3 Strain identification by MALDI‐TOF MS and 16S rRNA gene sequencing

The MALDI‐TOF MS protein analysis consisted of picking an isolated colony and then depositing twelve distinct deposits on a MTP 96 MALDI‐TOF target plate (Bruker Daltonics, Leipzig, Germany) to be analyzed. 2 μl of a matrix solution (saturated solution of α‐cyano‐4‐hydroxycinnamic acid diluted in 50% acetonitrile and 2.5% of tri‐fluoro‐acetic acid) was added on each spot. Measurements and proteomic analysis of the isolate were carried out with a Microflex spectrometer (Bruker) as previously described (Seng et al., 2009). Protein spectra were imported into the MALDI BioTyper software (version 2.0, Bruker) and analyzed by standard pattern matching (with default parameter settings) against the spectra of the Bruker database (constantly incremented with our new spectra). A score >1.9 enabled the identification at the species level and a score < 1.7 did not enable any identification. Sequencing the 16S rRNA gene is needed to achieve the identification if the bacterium is not referenced in the database. The 16S rRNA gene amplification and sequencing were performed as previously described (Morel et al., 2015). For similarity level thresholds of 98.65% and 95%, a new species or a new genus was suggested, respectively, as proposed by Kim, Oh, Park, & Chun, (2014).

### 2.4 Phylogenetic tree

A custom python script was used to automatically retrieve all species from the same order of the new genus and download 16S sequences from NCBI, by parsing NCBI eutils results and NCBI taxonomy page. It only keeps sequences from type strains. In case of multiple sequences for one type strain, it selects the sequence obtaining the best identity rate from the BLASTn alignment with our sequence. The script then separates 16S sequences in two groups: one containing the sequences of strains from the same family (group a) and one containing the others (group b). It finally only keeps the 15 closest strains from group a and the closest one from group b. If it is impossible to get 15 sequences from group a, the script selects more sequences from group b to get at least nine strains from both groups.

### 2.5 Growth conditions

Growth of the strain AT8^T^ was tested under anaerobic and microaerophilic conditions using GENbag anaer and GENbag microaer systems, respectively (bioMérieux, Marcy l'Etoile, France), and in aerobic conditions, with or without 5% CO_2_. Different temperatures (25, 30, 37, 45°C) were tested to determine the optimal growth of the strain AT8^T^. Optimal salt concentration required for growth was determined by growing the strain at 0, 0.5, 1,and 1.5% of NaCl. The optimal pH for growth was determined by testing different pH: 5, 6, 6.5, 7, 7.5, 8, and 8.5.

### 2.6 Morphological, biochemical, and antibiotic susceptibility tests

Sporulation assay was done by a thermic shock at 60°C for 20 min follow by a subculture on 5% sheep blood‐enriched Columbia agar medium (bioMérieux). Using a DM1000 photonic microscope (Leica Microsystems, Nanterre, France) with a100X objective lens, the motility of the strain from a fresh culture was observed. The colony's surface was observed on a 5% sheep blood agar culture medium after 24‐hr’ incubation at 37°C. In order to observe the cells morphology, they were fixed with 2.5% glutaraldehyde in 0.1mol/L cacodylate buffer for at least 1 hr at 4°C. A drop of the cell suspension was deposited for approximately 5 min on glow‐discharged formvar carbon film on 400 mesh nickel grids (FCF400‐Ni, EMS). The grids were dried on blotting paper and cells were negatively stained for 10 s with 1% ammonium molybdate solution in filtered water at RT. Electron micrographs were acquired with a Tecnai G^20^ Cryo (FEI) transmission electron microscope operated at 200 keV. The gram coloration was performed using the color Gram 2 kit (bioMérieux) and observed using a DM1000 photonic microscope (Leica Microsystems).

For the biochemical characterization assays, available API ZYM and API 50 CH strips (bioMérieux) were performed according to the manufacturer's instructions. Cellular fatty acid methyl ester (FAME) analysis was performed by gaz chromatography/mass spectrometry (GC/MS). Two samples were prepared with approximately 20 mg of bacterial biomass per tube harvested from several culture plates. FAME were prepared as described by Sasser, (2006). GC/MS analyses were carried out as described before (Dione et al., 2016). Briefly, FAME were separated using an Elite 5‐MS column and monitored by mass spectrometry (Clarus 500 ‐ SQ 8 S, Perkin Elmer, Courtaboeuf, France). Spectral database search was performed using MS Search 2.0 operated with the Standard Reference Database 1A (NIST, Gaithersburg, USA) and the FAMEs mass spectral database (Wiley, Chichester, UK).

Susceptibility to antibiotics was tested using Müller Hinton agar medium (bioMérieux) according to EUCAST 2015 recommendations (http://www.eucast.org). The following antibiotics were tested: doxycycline, cefoxitin, ciprofloxacin, clindamycin, erythromycin, fosfomycin, linezolid, oxacillin, penicillin, pristinamycin, rifampicin, teicoplanin, trimethoprim‐sulfamethoxazole, vancomycin, colistin, and metronidazole.

### 2.7 DNA extraction and genome sequencing and assembly

Strain AT8^T^ was cultured on ten petri dishes with 5% sheep blood Columbia agar. Genomic DNA (gDNA) of strain AT8^T^ was extracted in two steps : a mechanical treatment was first performed by glass beads acid washed (G4649‐500 g Sigma) using a FastPrep BIO 101 instrument (Qbiogene, Strasbourg, France) at maximum speed (6.5) for 3 × 30s. Then after a 2 hr lysozyme incubation at 37°C, DNA was extracted on the EZ1 biorobot (Qiagen) with EZ1 DNA tissues kit. The elution volume is 50 μl. gDNA was quantified by a Qubit assay with the high sensitivity kit (Life technologies, Carlsbad, CA, USA) to 29.1 ng/μl.

The gDNA of strain AT8^T^ was sequenced on MiSeq Technology (Illumina Inc, San Diego, CA, USA) with the mate pair strategy. The gDNA was barcoded in order to be mixed with 11 other projects with the Nextera Mate Pair sample prep kit (Illumina). The mate pair library was prepared with 1.5 μg of gDNA using the Nextera mate pair Illumina guide. The gDNA sample was simultaneously fragmented and tagged with a mate pair junction adapter. The pattern of the fragmentation was validated on an Agilent 2100 BioAnalyzer (Agilent Technologies Inc, Santa Clara, CA, USA) with a DNA 7500 labchip. The DNA fragments ranged in size from 1.5 kb up to 11 kb with an optimal size at 6.593 kb. No size selection was performed and 395 ng of tagmented fragments were circularized. The circularized DNA was mechanically sheared to small fragments with an optimal at 983 bp on the Covaris device S2 in T6 tubes (Covaris, Woburn, MA, USA). The library profile was visualized on a High Sensitivity Bioanalyzer LabChip (Agilent Technologies Inc, Santa Clara, CA, USA) and the final concentration library was measured at 23.68 nmol/L. The libraries were normalized at 3 nmol/L and pooled. After a denaturation step and dilution, the pool of libraries was loaded onto the reagent cartridge and then onto the instrument along with the flow cell. Automated cluster generation and sequencing run were performed in a single 2 × 251‐bp run. Genome assembly was performed in pipelines that allow us to make assembly with different assembly's softwares, on trimmed or not trimmed data. Velvet (Zerbino & Birney, 2008), Spades (Bankevich et al., 2012), and Soap Denovo (Luo et al., 2012) were used on trimmed (MiSeq software and Trimmomatic (Bolger, Lohse, & Usadel, 2014) software) and on not trimmed data (only MiSeq software). For each of the six assemblies performed, GapCloser (Luo et al., 2012) was used to reduce gaps. Then contamination with Phage Phix was identified (blastn against Phage Phix174 DNA sequence) and eliminated. At the end, scaffolds with size under 800 bp were removed and scaffolds with depth value lower than 0.25 of the mean depth are removed (identified as possible contaminant). The best assembly is selected on different criterion (number of scaffolds, N50, number of N). For strain AT8^T^, the best assembly was obtained with Spades and a coverage of 349. Default parameters were used, together with those specific parameters: –careful and kmer 77,99,127.

Total information of 10 Gb was obtained from a 690 K/mm^2^ cluster density with a cluster passing quality control filters of 94.5% (16,542,000 passing filter paired reads). Within this run, the index representation for strain AT8^T^ was determined to 7.36%. The 1,218,050 paired reads were trimmed then assembled in nine scaffolds.

### 2.8 Genome annotation and comparison

The prediction of open reading frames (ORFs) was performed by Prodigal (http://prodigal.ornl.gov/) with default parameters but the predicted ORFs were excluded if they were spanning a sequencing gap region (containing N). The predicted bacterial protein sequences were searched against the clusters of orthologous groups (COG) (Galperin, Makarova, Wolf, & Koonin, 2015) using BLASTP (E‐value 1e^−03^, coverage 0.7, and identity percent 30%). If no hit was found, it was searched against the NR database using BLASTP with E‐value of 1e^−03^ coverage 0.7 and identity percent of 30%, and if the sequence length was smaller than 80 amino acids, we used an E‐value of 1e^−05^. tRNA genes were found by the tRNAScanSE tool, whereas ribosomal RNAs were found using RNAmmer (Lagesen et al., 2007; Lowe & Eddy, 1997). Using Phobius, the lipoprotein signal peptides and the number of transmembrane helices were predicted (Käll, Krogh, & Sonnhammer, 2004). ORFans were identified if all the BLASTP performed did not give positive results (*E*‐value smaller than 1e^−03^ for ORFs with sequence size larger than 80 aa or *E*‐value smaller than 1e^−05^ for ORFs with sequence length smaller 80 aa). The XEGEN software (Phylopattern) allowed us to automatically retrieve genomes from the 16S RNA tree (Gouret, Thompson, & Pontarotti, 2009). For each selected species, the complete genome sequence, proteome sequence, and Orfeome sequence were retrieved from the FTP of NCBI. The proteomes were analyzed with proteinOrtho (Lechner et al., 2011). Then for each couple of genomes, a similarity score (mean value of nucleotide similarity between all couple of orthologues between the two genomes studied) was computed by AGIOS software (Average Genomic Identity Of gene Sequences) (Ramasamy et al., 2014). An annotation of all proteome was done to determine the predicted genes functional classes’ distribution according to the clusters of orthologous groups of proteins. The Multi‐Agent software system DAGOBAH, which includes Figenix libraries for provide pipeline analysis and Phylopattern for tree manipulation, was used to perform the annotation and comparison processes (Gouret et al., 2011). Genome‐to‐Genome Distance Calculator (GGDC) analysis was performed using the GGDC web server as previously reported (Meier‐Kolthoff, Auch, Klenk, & Göker, 2013).

## 3 Results

### 3.1 Phenotypic and biochemical features

Strain AT8^T^ presents small, smooth, shiny, circular colonies with a diameter of 2**–**5 mm. Strain AT8^T^ is gram‐positive, nonmotile, and nonspore forming (Fig. S1). Cells are rod‐shaped with a mean diameter of 0.8–1.2 μm (Fig. S2). Growth was obtained at temperatures ranging from 28 to 55°C and pH ranging from 6.5 to 8 with an optimum growth at 37°C and pH 7 after 48 hr incubation. The isolate did not require NaCl for growth; an optimal growth was observed at 0 or 0.5% of NaCl. No growth was observed at 1 and 1.5% of NaCl. Strain AT8^T^ was strictly anaerobic and did not grow in aerobic or 5% CO_2_ atmospheres. The principal characteristics of the strain and classification are present in Table 1.

**Table 1:** Classification and general features of *Hugonella massiliensis* strain AT8^T^ according to the MIGS recommendations (Field et al., 2008)

MIGS ID	Property	Term	Evidence code^a^
	Current classification	Domain: *Bacteria*	TAS (Woese, Kandler, & Wheelis, 1990)
		Phylum: *Actinobacteria*	TAS (Garrity & Holt, 2001)
		Class: *Coriobacteriia*	TAS (Skerman, McGowan, & Sneath, 1980)
		Order: *Eggerthellales*	TAS (Wade et al., 1999)
		Family: *Eggerthellaceae*	TAS (Moore, Cato, & Holdeman, 1971)
		Genus: *Hugonella*	IDA
		Species: *Hugonella massiliensis*	IDA
		Type strain: AT8^T^	IDA
	Gram stain	Positive	IDA
	Cell shape	Cocci	IDA
	Motility	Nonmotile	IDA
	Sporulation	nonspore forming	IDA
	Temperature range	Mesophile	IDA
	Optimum temperature	37°C	IDA
	pH	pH 6.5 to 8	
	Optimum pH	7	
MIGS‐6.3	Salinity	0.5 to 1.5%	IDA
	Optimum salinity	0‐0.5% NaCl	IDA
MIGS‐22	Oxygen requirement	Strictly anaerobic	IDA
	Carbon source	Unknown	IDA
	Energy source	Unknown	IDA
MIGS‐6	Habitat	Human gut	IDA
MIGS‐15	Biotic relationship	Free living	IDA
	Pathogenicity	Unknown	NAS
	Biosafety level	Unknown	IDA
MIGS‐14	Isolation	Human feces	IDA
MIGS‐4	Geographic location	France	IDA
MIGS‐5	Sample collection time	2012	IDA
MIGS‐4.3	Depth	Surface	IDA
MIGS‐4.4	Altitude	0 m above sea level	IDA

a Evidence codes ‐ IDA: Inferred from Direct Assay; TAS: Traceable Author Statement (i.e., a direct report exists in the literature); NAS: Nontraceable Author Statement (i.e., not directly observed for the living, isolated sample, but based on a generally accepted property for the species, or anecdotal evidence). These evidence codes are from http://www.geneontology.org/GO.evidence.shtml of the Gene Ontology project (Ashburner et al., 2000). If the evidence is IDA, then the property was directly observed for a live isolate by one of the authors or an expert mentioned in the acknowledgments.

Strain AT8^T^ has no catalase and oxidase activities. Using an API ZYM strip, positive reactions were observed for esterase (C4), esterase lipase (C8), naphthol‐AS‐BI‐phosphohydrolase, lipase (C14), leucine arylamidase, valine arylamidase, trypsin, α‐chymotrypsin, β‐galactosidase, N‐acetyl‐ β‐glucosaminidase, α‐galactosidase, β‐glucuronidase, α‐glucosidase, β‐glucosidase, α‐fucosidase, α‐mannosidase, and negative reaction was observed for alkaline phosphatase. An API 50CH strip showed negative reactions for arbutin, salicin, D‐maltose, D‐fructose, D‐sucrose, D‐raffinose, glycerol, erythritol, D‐ribose, D‐xylose, L‐xylose, D‐adonitol, methyl‐βD‐xylopyranoside, D‐glucose, D‐galactose, D‐lactose, L‐sorbose, L‐rhamnose, dulcitol, inositol, D‐mannitol, D‐sorbitol, methyl‐αD‐mannopyranoside, methyl‐αD‐glucopyranoside, D‐cellobiose, D‐melibiose, D‐trehalose, D‐melezitose, starch, glycogen, xylitol, gentiobiose, D‐turanose, D‐lyxose, D‐tagatose, D‐fucose, L‐fucose, D‐arabitol, L‐arabitol, and potassium gluconate; and positive reactions for D‐mannose, amygdalin, N‐acetyl‐glucosamine, esculin ferric citrate, D‐arabinose, inulin, potassium 2‐ketogluconate, and potassium 5‐ketogluconate. The phenotypic characteristics of Strain AT8^T^ were compared with the most closely related species (Table 2). The most abundant fatty acids were saturated structures: hexadecanoic acid C16:0 (35%) and tetradecanoic acid C14:0 (31%). Several branched fatty acids were also detected and only three unsaturated structures were described (Table 3).

**Table 2:** Differential characteristics of *Hugonella massiliensis* strain AT8^T^, *Eggerthella lenta* (Kageyama, Benno, & Nakase, 1999), *Denitrobacterium detoxificans* (Anderson, Rasmussen, Jensen, & Allison, 2000)*, Slackia exigua* (Kim et al., 2010), *Slackia heliotrinireducens* (Lanigan, 1976), *Gordonibacter pamelaeae* (Würdemann et al., 2009), *Adlercreutzia equolifaciens* (Maruo, Sakamoto, Ito, Toda, & Benno, 2008). na: Nonavailable data

Properties	*Hugonella massiliensis*	*Eggerthella lenta*	*Denitrobacterium detoxificans*	*Slackia exigua*	*Slackia heliotrinireducens*	*Gordonibacter pamelaeae*	*Adlercreutzia equolifaciens*
Cell diameter (μm)	0.8–1.2	0.4–0.8	0.5–1 × 1 –1.5	0.5–1.0	0.8–1.2	0.6–1.01	0.6–1.5
Oxygen requirement	−	–	–	+	–	+	–
Gram stain	+	+	+	+	+	+	+
Salt requirement	−	na	na	–	+	–	na
Motility	−	–	–	–	–	+	–
Endospore formation	−	−	−	−	−	–	–
Indole	−	−	−	−	−	−	na
Production of							
Alkaline phosphatase	−	na	−	−	na	na	na
Catalase	−	−	−	−	−	+	na
Oxidase	−	−	−	−	−	−	na
Nitrate reductase	−	+	+	+	+	+	−
Urease	−	na	na	−	−	na	−
β‐galactosidase	−	na	na	−	na	na	−
N‐acetyl‐glucosamine	+	na	na	na	na	na	na
Acid from							
L‐Arabinose	+	−	na	−	−	−	+
Ribose	−	−	−	−	−	−	na
Mannose	+	−	na	−	−	−	−
Mannitol	−	−	−	na	na	−	+
Sucrose	−	na	na	na	−	na	na
D‐glucose	−	−	−	−	−	−	−
D‐fructose	−	−	−	−	na	−	na
D‐maltose	−	−	na	na	na	−	na
D‐lactose	−	na	na	na	na	na	na
Habitat	Human gut	Human gut	Ruminal microbes	Human gut	Human gut	Human gut	Human gut

**Table 3:** Cellular fatty acid composition (%) of *Hugonella massiliensis* strain AT8^T^

Fatty acids	Name	Mean relative % a
C16:0	Hexadecanoic acid	35.0 ± 1.5
C14:0	Tetradecanoic acid	30.9 ± 0.5
C15:0 anteiso	12‐methyl‐tetradecanoic acid	7.3 ± 0.6
C18:1n9	9‐Octadecenoic acid	6.5 ± 1.5
C15:0 iso	13‐methyl‐tetradecanoic acid	6.3 ± 0.8
C18:0	Octadecanoic acid	5.7 ± 1.7
C14:0 iso	12‐methyl‐Tridecanoic acid	3.4 ± 0.4
C15:0	Pentadecanoic acid	1.9 ± 0.1
C18:2n6	9,12‐Octadecadienoic acid	1.5 ± 0.1
C18:1n7	11‐Octadecenoic acid	1.5 ± 0.5
C17:0	Heptadecanoic acid	TR

a Mean peak area percentage; TR = trace amounts <1%.

Antimicrobial susceptibility testing demonstrates that the strain AT8^T^ was susceptible to cefoxitin, ciprofloxacin, clindamycin, fosfomycin, linezolid, oxacillin, penicillin, pristinamycin, rifampicin, teicoplanin, trimethoprim‐sulfamethoxazole, vancomycin, and metronidazole, and resistant to doxycycline, erythromycin, and colistin.

### 3.2 Strain identification and phylogenetic analysis

**Figure 1:**
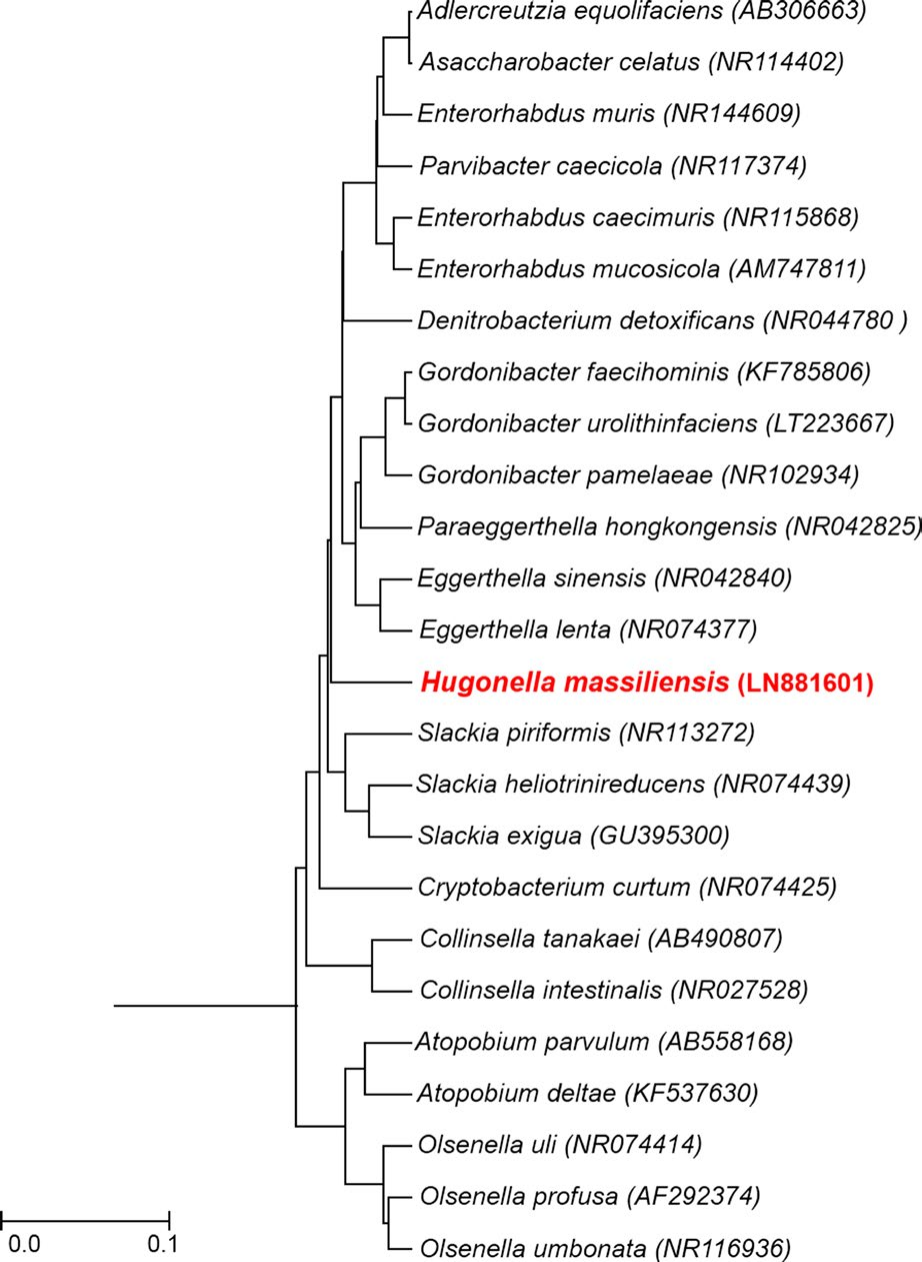
Phylogenetic tree highlighting the position of *Hugonella massiliensis* strain AT8^T^ relative to other close strains. Sequences were aligned using Muscle v3.8.31 with default parameters and phylogenetic inferences were obtained using neighbor‐joining method with 500 bootstrap replicates, within MEGA6 software. The scale bar represents a 0.1% nucleotide sequence divergence

**Figure 2:**
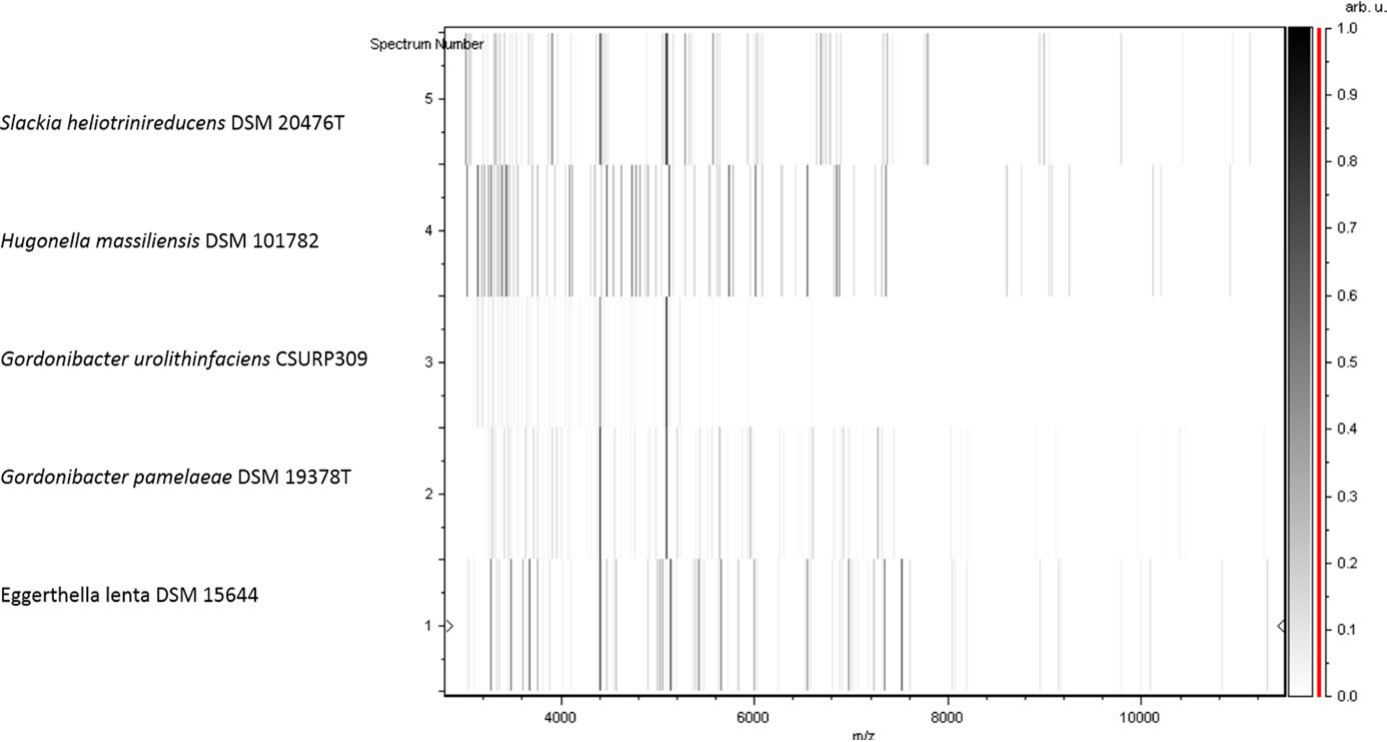
Gel view comparing *Hugonella massiliensis* strain AT8^T^ to other closely related species. The gel view displays the raw spectra of strain AT8^T^ of loaded spectrum files arranged in a pseudo‐gel like look. The *x*‐axis records the m/z value. The left *y*‐axis displays the running spectrum number originating from subsequent spectra loading. The peak intensity is expressed by a gray scale scheme code. The right *y*‐axis indicates the relation between the color of a peak and its intensity, in arbitrary units. Displayed species are indicated on the left

The spectra of strain AT8^T^ obtained by MALDI‐TOF MS did not match with any strains in our database (Bruker, continuously incremented with our data) suggesting that our isolate could be a new isolate. We added the spectrum from strain AT8^T^ to URMITE database (Figure S3) (http://www.mediterranee-infection.com/article.php?laref=256&titre=urms-database). PCR‐based identification of the16S rDNA of strain AT8^T^ (Accession number: LN881601) demonstrated 92% of 16S rRNA gene sequence similarity with the reference *Eggerthella lenta* (DSM 2243), the phylogenetically closest validated species (Figure 1). This value is under the threshold that allows the identification of a new genus, as established by Kim et al., (2014). Consequently, strain AT8^T^ is considered as the type strain of the first isolate of a new genus named *Hugonella* gen. nov., for which *Hugonella massiliensis* sp. nov strain AT8^T^ is the type strain. Finally, the gel view showed the mass spectra's differences with other closely related genera of *Eggerthellacae* family (Figure 2).

### 3.3 Genome properties

**Figure 3:**
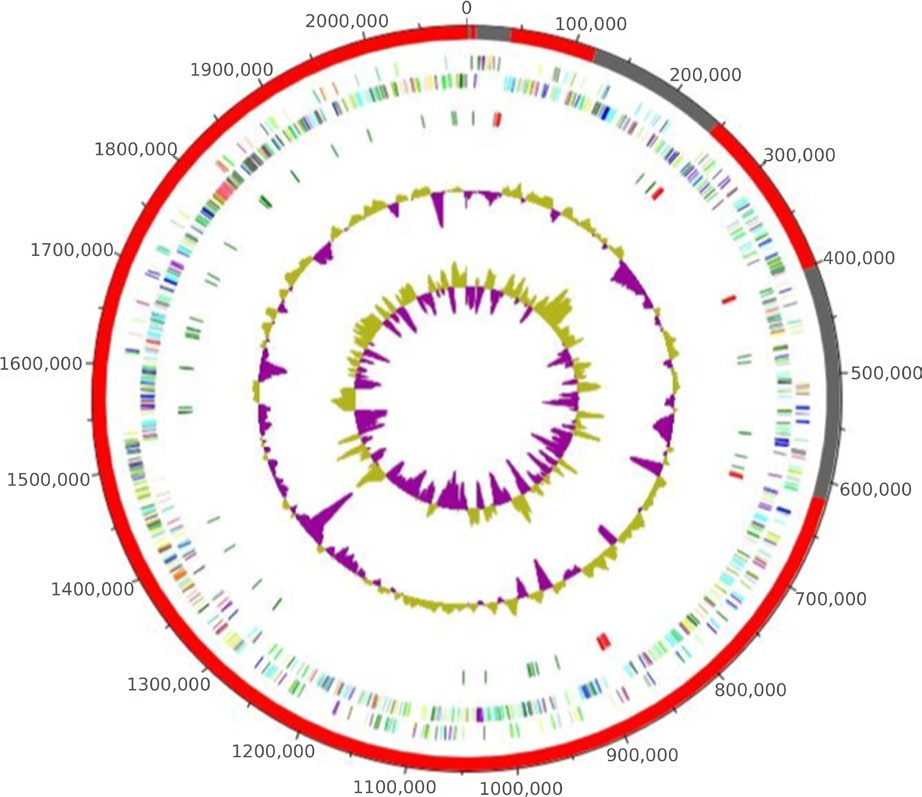
Graphical circular map of the chromosome. From outside to the center: Genes on the forward strand colored by Clusters of Orthologous Groups (COG) categories (only gene assigned to COG), genes on the reverse strand colored by COG categories (only gene assigned to COG), RNA genes (tRNAs green, rRNAs red), G+C content, and G+C skew

The genome of *H. massiliensis* strain AT8^T^ (Accession number: FAUL00000000) contains 2,091,845 bp with 63.46% of G+C content (Table 4, Figure 3) and is composed of nine scaffolds with 12 contigs. The draft genome was shown to encode 1,849 predicted genes, among which 1,781 were protein‐coding genes, and 68 were RNAs (six genes are 5S rRNA, six genes are 16S rRNA, six genes are 23S rRNA, 50 genes are tRNA genes). A total of 1,438 genes (80.74%) were assigned as putative function (by cogs or by NR blast). Ninety genes (5.05%) were identified as ORFans. The remaining genes were annotated as hypothetical proteins (201 genes = 11.29%) (Table 4). Table 5 represents the gene's distribution into clusters of orthologous groups (COGs).

**Table 4:** The genome nucleotide content and gene count levels of *Hugonella massiliensis* strain AT8^T^

Attribute	Genome (total)	
	Value	% of total^a^
Size (bp)	2,091,845	100
G+C content (%)	1,326,454	63.46
Coding region (bp)	1,844,967	88.19
Total genes	1,849	100
RNA genes	68	3.69
Protein‐coding genes	1,781	100
Genes with function prediction	1,438	80.74
Genes assigned to COGs	1,242	69.73
Genes with peptide signals	156	8.75
CRISPR repeats	0	0
ORFn genes	90	5.05
Genes associated with PKS or NRPS	8	0.44
No. of antibiotic‐resistant genes	0	0

a The total is based on either the size of the genome in base pairs or the total number of protein‐coding genes in the annotated genome.

**Table 5:** Number of genes associated with the 25 general COG functional categories

Code	Value	% of total	Description
[J]	154	8.646828	Translation
[A]	0	0	RNA processing and modification
[K]	72	4.0426726	Transcription
[L]	64	3.593487	Replication, recombination, and repair
[B]	0	0	Chromatin structure and dynamics
[D]	21	1.1791129	Cell cycle control, mitosis, and meiosis
[Y]	0	0	Nuclear structure
[V]	22	1.2352611	Defense mechanisms
[T]	49	2.7512634	Signal transduction mechanisms
[M]	56	3.1443012	Cell wall/membrane biogenesis
[N]	12	0.6737788	Cell motility
[Z]	0	0	Cytoskeleton
[W]	7	0.39303765	Extracellular structures
[U]	19	1.0668164	Intracellular trafficking and secretion
[O]	69	3.8742278	Posttranslational modification, protein turnover, chaperones
[X]	9	0.5053341	Mobilome: prophages, transposons
[C]	147	8.25379	Energy production and conversion
[G]	61	3.425042	Carbohydrate transport and metabolism
[E]	155	8.702975	Amino acid transport and metabolism
[F]	59	3.3127456	Nucleotide transport and metabolism
[H]	80	4.4918585	Coenzyme transport and metabolism
[I]	70	3.930376	Lipid transport and metabolism
[P]	74	4.154969	Inorganic ion transport and metabolism
[Q]	23	1.2914094	Secondary metabolites biosynthesis, transport, and catabolism
[R]	117	6.569343	General function prediction only
[S]	52	2.919708	Function unknown
_	539	30.263897	Not in COGs

The total is based on the total number of protein‐coding genes in the annotated genome.

### 3.4 Genome comparison

**Figure 4:**
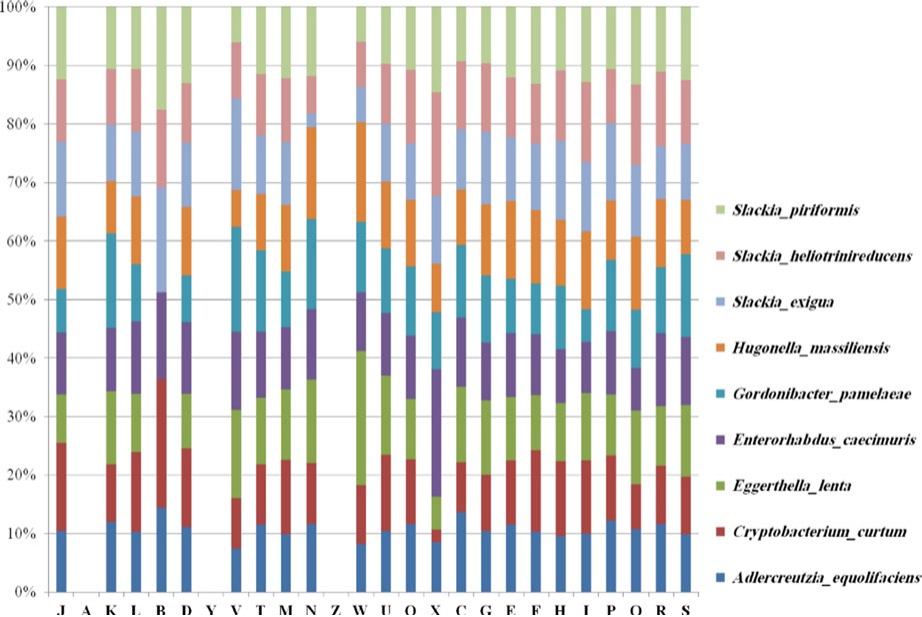
Distribution of functional classes of predicted genes according to the clusters of orthologous groups of proteins of *Hugonella massiliensis* strain AT8^T^ and other close related species

We made some comparisons with the closest annotated sequenced genomes currently available: *Slackia piriformis* ADMD00000000.1, *Slackia exigua* ACUX00000000.2, *Gordonibacter pamelaeae* FP929047.1, *Enterorhabdus caecimuris* ASSY00000000.1, *Slackia heliotrinireducens* CP001684.1, *Adlercreutzia equolifaciens* AP013105.1, *Eggerthella lenta* CP001726.1, *Cryptobacterium curtum* CP001682.1, and *Denitrobacterium detoxificans* (CP011402.1) (Table 6). The strain AT8^T^ has a smaller draft genome sequence than those of *S. piriformis*,* S. exigua*,* A. equolifaciens*,* G. pamelaeae*,* E. caecimuris*,* S. heliotrinireducens, E. lenta,* and *D. detoxificans* (respectively, 2.09, 2.12, 2.10, 2.86, 3.61, 2.96, 3.17, 3.63 Mb and 2.45) but larger than those of *C. curtum* (1.62 Mb). The G+C content of *H. massiliensis* (63.46%) is larger than those of *S. piriformis*,* S. exigua*,* A. equolifaciens*,* C. curtum, S. Heliotrinireducens,* and *D. detoxificans* (57.68, 62.15, 63.46, 50.91, 60.21%, and 59.5%, respectively), but smaller than those of *G. pamelaeae, E. caecimuris,* and *E. lenta* (65.48, 64.13, and 64.20%, respectively). The gene content of *H. massiliensis* is smaller than those of *S. piriformis*,* S. exigua*,* A. equolifaciens*,* G. pamelaeae*,* E. caecimuris*,* S. heliotrinireducens, E. lenta,* and *D. detoxificans* (1,781, 1,799, 2,029, 2,281, 2,027, 2,455, 2,765, 3,070, 1,989, respectively), but larger than those of *C. curtum* (1,357) (Table 6). The distribution of genes into COG categories is identical in all genomes compared (Figure 4). Calculation of the Average Genomic Identity of Orthologous gene Sequences (AGIOS) shows that *H. massiliensis* shared 800, 757, 854, 482, 796, 830, 922, and 844 orthologous genes with *S. piriformis*,* S. exigua*,* S. heliotrinireducens, G. pamelaeae*,* C. curtum, E. caecimuris, E. lenta,* and *A. equolifaciens,* respectively (Table 7). The AGIOS values ranged from 53.43% to 81.88% among species with standing in nomenclature, except *H. massiliensis*. When *H. massiliensis* was compared to the other closest species, the AGIOS values ranged from 57.10% with *S. exigua* to 71.59% with *G. pamelaeae* (Table 7). Among species with standing in nomenclature, dDDH values ranged from 19.4% to 29.4%. dDDH values between strain AT8^T^ and compared species ranged from 19.8% with *A. equolifaciens* to 22% with *S. piriformis* and *C. curtum* (Table 8).

**Table 6:** Genome comparison between *Hugonella massiliensis* strain AT8^T^ and closely related species

Name of organisms	INSDC	Size (Mb)	G+C (%)	Total Genes
*Hugonella massiliensis* strain AT8^T^	FAUL00000000	2.091	63.46	1,781
*Eggerthella lenta* DSM2243	CP001726.1	3.63	64.2	3,070
*Cryptobacterium curtum* DSM15641	CP001682.1	1.62	50.91	1,357
*Gordonibacter pamelaeae* strain 7‐10‐1‐b	FP929047.1	3.61	65.48	2,027
*Adlercreutzia equolifaciens* FJC‐B9	AP013105.1	2.86	63.46	2,281
*Slackia piriformis* YIT_12062	ADMD00000000.1	2.12	57.68	1,799
*Slackia exigua* ATCC700122	ACUX00000000.2	2.09	62.15	2,029
*Enterorhabdus caecimuris* B7	ASSY00000000.1	2.96	64.13	2,455
*Slackia heliotrinireducens* DSM20476	CP001684.1	3.16	60.21	2,765
*Denitrobacterium detoxificans* DSM 21843	CP011402.1	2.45	59.5	1,989

**Table 7:** The number of orthologous protein shared between genomes (upper right), average percentage similarity of nucleotides corresponding to orthologous proteins shared between genomes (lower left), and numbers of proteins per genome (bold)

	*Hugonella massiliensis*	*Slackia piriformis*	*Slackia exigua*	*Slackia heliotrinireducens*	*Gordonibacter pamelaeae*	*Cryptobacterium curtum*	*Enterorhabdus caecimuris*	*Eggerthella lenta*	*Adlercreutzia equolifaciens*	Denitrobacterium detoxificans
*Hugonella massiliensis*	**1,781**	59.31	57.11	68.36	71.60	67.79	60.90	70.37	844	847
*Slackia piriformis*	800	**1,799**	798	870	528	713	892	983	911	805
*Slackia exigua*	757	61.71	**2,029**	825	437	736	761	844	789	786
*Slackia heliotrinireducens*	854	60.54	57.58	**2,765**	499	725	864	948	878	867
*Gordonibacter pamelaeae*	482	61.32	57.98	70.12	**2,027**	434	584	715	587	629
*Cryptobacterium curtum*	796	5743	53.43	63.75	65.51	**1,357**	730	834	745	785
*Enterorhabdus caecimuris*	830	59.30	62.29	59.98	63.70	56.45	**2,455**	1,076	1,143	894
*Eggerthella lenta*	922	60.55	57.88	69.33	81.88	64.70	62.93	**3,070**	1,108	940
*Adlercreutzia equolifaciens*	69.59	59.60	57.02	68.64	74.63	63.90	67.426	73.39	**2,281**	917
Denitrobacterium detoxificans	72 .5	67.69	68.26	68.56	71.11	68.49	68.71	70.08	68.74	**1,989**

**Table 8:** Pairwise comparison of *Hugonella massiliensis* with other species using GGDC, formula 2 (DDH estimates based on identities/HSP length)* upper right

	*Hugonella massiliensis*	*Cryptobacterium curtum*	*Gordonibacter pamelaeae*	*Eggerthella lenta*	*Enterorhabdus caecimuris*	*Adlercreutzia equolifaciens*	*Slackia piriformis*	*Slackia exigua*	*Slackia heliotrinireducens*	Denitrobacterium detoxificans
*Hugonella massiliensis*	**100 ± 00**	22 ± 2.35	20.5 ± 2.32	20.1 ± 2.31	20.6 ± 2.32	19.8 ± 2.3	22 ± 2.35	20.8 ± 2.33	20.3 ± 2.32	21.8 ± 2.35
*Cryptobacterium curtum*		**100 ± 00**	25.4 ± 2.41	22.4 ± 2.36	22.5 ± 2.36	24.1 ± 2.39	22.6 ± 2.36	23.6 ± 2.38	26.8 ± 2.42	21.1 ± 2.30
*Gordonibacter pamelaeae*			**100 ± 00**	29.4 ± 2.44	22.4 ± 2.36	22.4 ± 2.36	20.8 ± 2.33	20.3 ± 2.31	19.8 ± 2.3	19.7 ± 2.30
*Eggerthella lenta*				**100 ± 00**	21.8 ± 2.35	21.5 ± 2.34	20.9 ± 2.33	20.1 ± 2.31	19.4 ± 2.29	20.2 ± 2.40
*Enterorhabdus caecimuris*					**100 ± 00**	25.6 ± 2.41	22.3 ± 2.36	19.8 ± 2.3	19.5 ± 2.29	19.4 ± 2.30
*Adlercreutzia equolifaciens*						**100 ± 00**	21.3 ± 2.34	19.4 ± 2.29	19.9 ± 2.3	19.5 ± 2.40
*Slackia piriformis*							**100 ± 00**	21 ± 2.33	21.5 ± 2.34	20.2 ± 2.30
*Slackia exigua*								**100 ± 00**	20.7 ± 2.32	20.3 ± 2.30
*Slackia heliotrinireducens*									**100 ± 00**	20.5 ± 2.30
**Denitrobacterium detoxificans**										**100 ± 00**

Bold value: Presents the comparison between the strain and itself

## 4 Discussion

Here, we used the culturomics approach to study the microbial diversity of the digestive tract of an obese subject in order to enrich the obese microbiota repertoire and establish a possible relationship between the biodiversity and morbid obesity in our patient. Here, we report the phenotypic characterization and genomic description of this new bacterial genus isolated from a stool specimen collected from a French female obese patient. Results reported here support the effectiveness of the culturomics procedure in the exploration of the bacterial diversity and the discovery of the remained unknown species to make their genomes available for eventual metagenomics study. In addition, culturing new bacteria permits the testing of culture conditions and provides information on antibiotic susceptibility. It is noted that this work does not present a medical interest of this strain but only broadens the knowledge about the microbial diversity of the human gut and extends the repertoire of the new species.

## 5 Conclusion

Based on phenotypic, phylogenetic, MALDI‐TOF, and genomic analyses, we formally propose the creation of the genus *Hugonella* gen. nov., including *Hugonella massiliensis* gen. nov., sp. nov currently the only cultivated species. Indeed, *H. massiliensis* strain AT8^T^ is only 92% 16S rRNA sequence similarity with *Eggerthella lenta* DSM2243 which gives it the status of a new genus. The strain has been isolated from a stool specimen of a morbidly obese French woman, as part of the culturomics study by anaerobic culture at 37°C. Several other bacterial species that remain undescribed were also isolated from different stool specimens using different culture conditions, suggesting that the human intestinal microbiota remains partially unknown and its diversity has yet to be fully explored.

### 5.1 Description of *Hugonella* gen. nov

*Hugonella* (Hu.gon.el'la, ML. dim. suffix *tella*; M.L. fem. n. *Hugonella* named after the French bacteriologist Perrine Hugon). Belongs to the family *Eggerthellaceae* within the phylum *Actinobacteria*. Cells are gram‐positive cocci, nonmotile, and nonspore forming. Mesophilic and do not require NaCl for growth. Cells do not produce catalase and oxidase. The most abundant fatty acids were saturated structures: hexadecanoic acid C16:0 (35%) and tetradecanoic acid C14:0 (31%). Several branched fatty acids were also detected and only three unsaturated structures were described. The DNA G+C content is approximately 63%. *Hugonella massiliensis* strain AT8^T^ the type strain was isolated from feces of a 51‐year‐old obese French woman (BMI 44.38 kg/m2).

### 5.2 Description of *Hugonella massiliensis* sp. nov

*Hugonella massiliensis* (mas.si.li.en'sis, L. fem. adj., *massiliensis* of Massilia, the Roman name of Marseille, France, where the type strain was isolated). Strictly anaerobic gram‐positive cocci, nonmotile, and nonspore forming. Cells diameter is of 0.8–1.2 μm. An optimal growth was observed at 37°C, pH 7, and NaCl is not required for growth. Colonies are smooth, shiny and measure 2–5 mm. No catalase and no oxidase activities were observed.

Using API strips, positive reactions were observed for esterase, esterase lipase, naphthol‐AS‐BI‐phosphohydrolase, D‐mannose, amygdalin, N‐acetyl‐glucosamine, esculin ferric citrate, D‐arabinose, inulin, potassium 2‐ketogluconate, potassium 5‐ketogluconate, lipase (C14), leucine arylamidase, valine arylamidase, trypsin, α‐chymotrypsin, β‐galactosidase, N‐acetyl‐ β‐glucosaminidase, α‐galactosidase, β‐glucuronidase, α‐glucosidase, β‐glucosidase, α‐fucosidase, and α‐mannosidase. Strain AT8^T^ was susceptible to cefoxitin, ciprofloxacin, clindamycin, fosfomycin, linezolid, oxacillin, penicillin, pristinamycin, rifampicin, teicoplanin, trimethoprim‐sulfamethoxazole, vancomycin, and metronidazole. The most abundant fatty acids were hexadecanoic acid C16:0 (35%) and tetradecanoic acid C14:0 (31%).

The genome of *H. massiliensis* strain AT8^T^ is 2,091,845 bp with 63.46% of G+C content. The 16S rRNA and genome sequences are deposited in EMBL‐EBI under accession numbers LN881601 and FAUL00000000, respectively. MALDI‐TOF Spectrum of strain AT8^T^ is available in: (http://mediterraner-infection.com/article.php?laref=256&titre=urms-database). The habitat of the microorganism is the human gut. The type strain AT8^T^ (=CSUR P2118 =  DSM 101782) was isolated from the stool specimen of an obese French individual as part of a culturomics study.

## Conflict of Interest

The authors declare no conflict of interest.
