# EXPRESSION OF CONCERN: *Hugonella massiliensis* gen. nov., sp. nov., Genome Sequence, and Description of a New Strictly Anaerobic Bacterium Isolated from the Human Gut

**DOI:** 10.1002/mbo3.70242

**Published:** 2026-02-19

## Abstract

**EXPRESSION OF CONCERN:** Z. Elsawi, A.H. Togo, M. Beye, G. Dubourg, C. Andrieu, N. Armstrong, M. Richez, F. di Pinto, F. Bittar, N. Labas, P.‐E. Fournier, D. Raoult, and S. Khelaifia, “*Hugonella massiliensis* gen. nov., sp. nov., Genome Sequence, and Description of a New Strictly Anaerobic Bacterium Isolated from the Human Gut,” by *Microbiology Open* 6, no. 4 (2017): e00458, https://doi.org/10.1002/mbo3.458.

This Expression of Concern is for the above article, published online on 21 March 2017 in Wiley Online Library (wileyonlinelibrary.com), has been issued by John Wiley & Sons Ltd. The Expression of Concern has been agreed due to questions raised about the study's adherence to French legal and ethical requirements for research involving human subjects. The investigation into these concerns is ongoing. Therefore, the journal has decided to issue an Expression of Concern to inform and alert readers.

