# Supplementary figures and images for: Hugonella massiliensis gen. nov., sp. nov., genome sequence, and description of a new strictly anaerobic bacterium isolated from the human gut
Source: Microbiologyopen. 2017 Mar 21;6(4):e00458. doi: 10.1002/mbo3.458 (PMC5552949; doi:10.1002/mbo3.458)

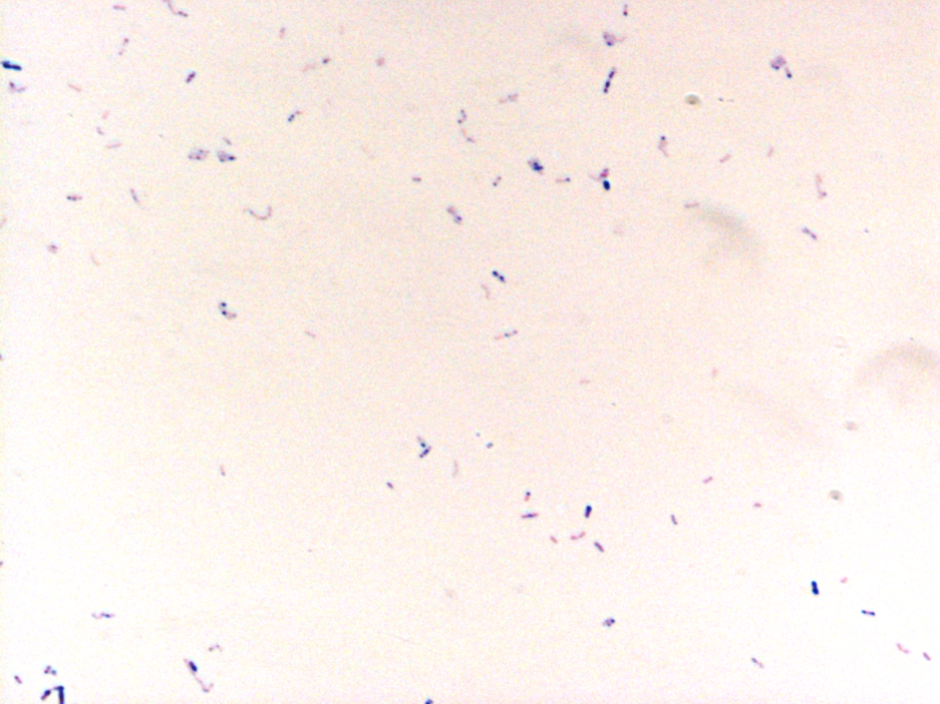


**Figure S1:** Gram staining of *Hugonella massiliensis* strain AT8T.

Supplement: Supplementary file 1 [file MBO3-6-na-s001.docx]

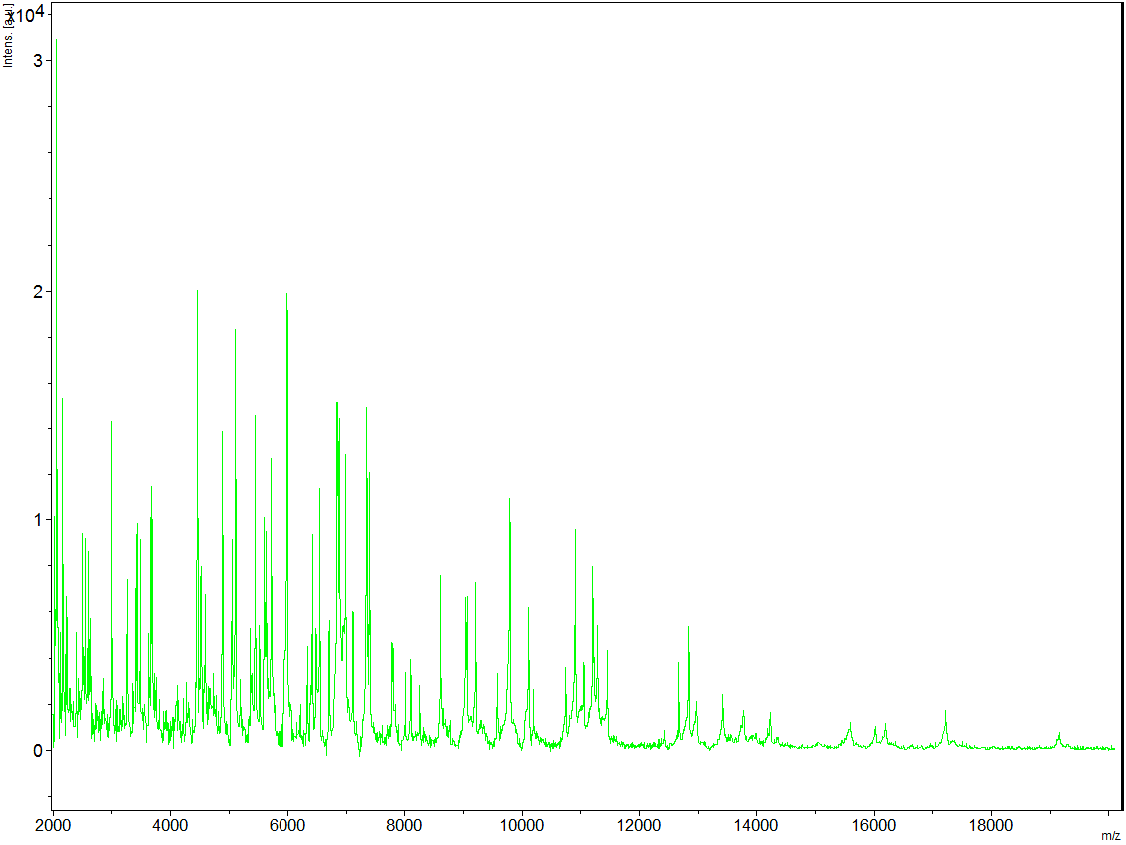


**Figure S3:** Reference mass spectrum (MALDI-TOF) from*Hugonella massiliensis* strain AT8T.

Supplement: Supplementary file 3 [file MBO3-6-na-s003.docx]
