# Supplementary material for: Hugonella massiliensis gen. nov., sp. nov., genome sequence, and description of a new strictly anaerobic bacterium isolated from the human gut
Source: Microbiologyopen. 2017 Mar 21;6(4):e00458. doi: 10.1002/mbo3.458 (PMC5552949; doi:10.1002/mbo3.458)
Supplement: Supplementary file 2 [file MBO3-6-na-s002.docx]

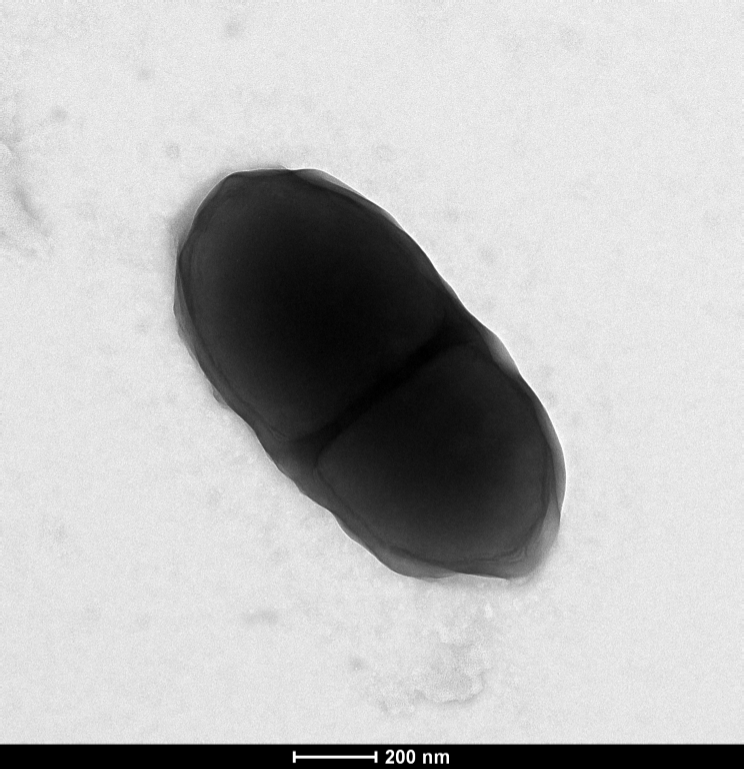


**Figure S2:** Transmission electron microscopy of *Hugonella massiliensis* strain AT8^T^ using a Tecnai G20 transmission electron microscope (FEI Company) at operating voltage of 200 keV. The scale bar represents 200 nm.
